# Proximal discrepancy in intrinsic atomic interaction arrests G2/M phase by inhibiting Cyclin B1/CDK1 to infer molecular and cellular biocompatibility of d-limonene

**DOI:** 10.1038/s41598-022-21364-4

**Published:** 2022-10-28

**Authors:** Deepa Mandal, Paritosh Patel, Suresh K. Verma, Bikash Ranjan Sahu, Tithi Parija

**Affiliations:** School of Biotechnology, KIIT Deemed to Be University, Bhubaneswar, Odisha 751024 India

**Keywords:** Biotechnology, Cell biology

## Abstract

The quest for different natural compounds for different biomedical applications especially in the treatment of cancer is at a high pace with increasing incidence of severity. d-limonene has been portrayed as one of the effective potential candidate centered to the context of breast cancer. The anticipation of its count as an effective biomedical agent required a detailed understanding of their molecular mechanism of biocompatibility. This study elucidates the mechanistic action of d-limonene channelized by the induction of apoptosis for controlling proliferation in breast cancer cells. The possible mechanism was explored through an experimental and computational approach to estimate cell proliferation inhibition, cell cycle phase distribution, apoptosis analysis using a flow cytometry, western blotting and molecular docking. The results showed reduced dose and time-dependent viability of MCF7 cells. The study suggested the arrest of the cell cycle at G2/M phase leading to apoptosis and other discrepancies of molecular activity mediated via significant alteration in protein expression pattern of anti-apoptotic proteins like Cyclin B1 and CDK1. Computational analysis showed firm interaction of d-limonene with Cyclin B1 and CDK1 proteins influencing their structural and functional integrity indicating the mediation of mechanism. This study concluded that d-limonene suppresses the proliferation of breast cancer cells by inducing G2/M phase arrest via deregulation of Cyclin B1/CDK1.

## Introduction

Globally, breast cancer is considered to be the most frequent malignancy in females^1^. Despite the ongoing clinical and laboratory research around the world, the rising incidences of this disease has led to many casualties^2,3^. Recently, many new drugs and natural products have been utilized for breast cancer treatment which reduced the mortality rate. However, the quest and demand for natural compounds still exists. d-limonene, a natural monocyclic and monoterpene compound which is prepared from the peel of citrus fruits, is one such compound which has gained lots of attention. Several pre-clinical studies have suggested the anticancer properties of this compound and has been reported to be safe and well tolerated on patients with solid tumors, based in the Phase I and phase II trials^4–7^.

Numerous studies have investigated the anti-cancer mechanism of d-limonene that revealed the induction of autophagy, apoptosis, angiogenesis etc., by interfering with multiple signaling pathways like NF-Kb, PI3K/AKT/MTOR and DNA damage repair^4,8–10^. Recently, it has been shown to target the oncogene PDIA3P1 that affects lipid metabolism immunity in LAUD cells^11^. The activation of both intrinsic and extrinsic pathway of apoptosis are also regulated by d-limonene. Apoptosis is efficient in removing abnormal cells arising due to DNA damage during developmental process. Regulated by several genes involved in several signaling pathways, deregulation of apoptosis leads to cancer via uncontrolled cell proliferation. Therefore, targeting apoptosis by natural compounds may restore the disrupted apoptotic pathway in cancer cells that might be a promising approach in the treatment of various cancers.

The evidence that modulation of the cell cycle can either prevent or promote an apoptotic response has led to a strong correlation between the cell cycle and apoptosis. Cell cycle is controlled by systematic regulated expressions of Cyclins and Cyclin-dependent-kinases. The Cyclin B1 plays an important role along with Cyclin dependent kinases 1(CDK1) during the transition of the cell cycle’s G2 phase to mitosis. Thus, deregulation of Cyclin B1 may contribute to uncontrolled cell proliferation^12^. Cyclin B1 overexpression has been reported in various cancers including breast cancer^12,13^. Therefore, inhibition of Cyclin B1 over-expression could be a therapeutic strategy to reduce the proliferation of abnormal cells and regulating apoptosis.

In view of the previous established mechanism, it can be hypothesized that d-limonene would have been controlling its activity by interfering with cellular processes. Though, previous efforts have been made to examine the mechanistic pathways for anti-cancer potential of this novel natural compound in terms of cell cycle regulation and apoptosis in various types of cancers, the issue is not clearly addressed in the context of breast cancer. To address this, the current study was done to evaluate the potential efficiency of d-limonene in inhibiting the growth of MCF7 breast cancer cells and understanding the molecular mechanism involved in the cell cycle and apoptosis regulation. Further, the significance of Cyclin B1 and CDK1 was highlighted for the first time in context to cell cycle arrest in d-limonene treated breast cancer cells.

## Materials and methods

### Cell culture

The human breast cancer cell line MCF 7 was purchased from the National Centre for Cell Science, Pune, India. The cells were maintained with DMEM medium (PAN Biotech) containing 10% fetal bovine serum (Gibco, Life Technologies, USA), supplemented with 100 U/mL penicillin, 100 μg/mL streptomycin (Gibco, Life Technologies, USA) and 1% (w/v) l-glutamine (PAN Biotech) and MCF10A cells (ATCC, CRL-10317) were cultured in DMEM/F12 medium supplemented with 5% horse serum, epidermal growth factor (20 ng/ml), insulin (10 µg/ml), hydrocortisone (0.5 mg/ml), cholera toxin (100 ng/ml) and penicillin/streptomycin at 37 °C in a humidified CO2 (5%) incubator. d-limonene was purchased from SIGMA, Life Science, USA^14^. Tamoxifen was purchased from MP-Biomedical.

### Cell survival assay (MTT)

The impact of d-limonene on MCF7 and MCF10A cells viability was analyzed using the 3-(4, 5-dimethylthiazol-2-yl) 2, 5-diphenyltetrazolium bromide (MTT) conversion assay as described in Razak et al.^15^*.* In brief, MCF7 and MCF10A cells were seeded at a density of 1 × 10^4^/ml onto 96 well tissue culture plate. After reaching nearly 60% of confluency, the cells were exposed to different concentration of d-limonene for 24, 48 and 72 for MCF7 cells, MCF10A cells for 48 h. d-limonene is solubilized in 0.1% dimethyl sulfoxide (DMSO) and was used as control for these assays. After 24 h of d-limonene exposure, 10ul of MTT (Merck Millipore, Massachusetts, USA), was put into each well and incubated for 4 h at 37 °C. Followed by incubation, media was aspirated and 100 μl of DMSO solution was poured into each well to dissolve the formazan crystals formed during the process. The optical density (OD) was measured at 570 nm with the help of an ELISA micro plate reader (BioTek, Vermont, USA). The relative percentage of viable cells was calculated using GraphPad Prism 6 software.

### Clonogenic assay

The clonogenic assay was performed according to the protocol mentioned in previous literature^16^. Around 5 × 10^2^ numbers of viable MCF7 cells were cultured in a 6 well tissue culture plate. The cells were treated with different concentrations (0.5 mM, 1 mM, 2 mM, 3 mM and 4 mM) of d-limonene for 24 h. Followed by incubation, the culture medium was changed and replaced with fresh medium and the d-limonene treated cells were allowed to grow for 5 to 6 doubling times. The plates were dried after removing the media and the colonies were stained with crystal violet (0.2%) for 1 h. Followed by staining, the cells were washed repeatedly to remove excess dye. Plates were dried in the air and stained colonies were counted manually by visualizing under the microscope. The data was analyzed, calculated for survivability and represented as percentage survival with respect to untreated MCF7 cells.

### Determination of apoptotic nuclei using DAPI

To check for apoptosis, nucleic acid staining was performed using DAPI as described by Reddy et al*.*^17^. Cells were seeded in 96 well tissue culture plates and allowed to grow for 24 h. Followed by incubation, they were treated with different concentrations of d-limonene (0.5 mM, 1 mM, 2 mM and 4 mM) and incubated for 24 h in standard cell culture conditions. After the treatment, cells were fixed in ice cold acetone and methanol solutions (1:1) at 4 °C for 30 min. Fixed cells were washed with chilled 1X PBS. Cells were covered with DAPI solution and incubated at 4 °C in the dark for five minutes. Excess of DAPI stain was taken out and cells were washed thrice with 1X PBS repeatedly. Then, the stained cells were observed under the fluorescence microscope.

### Apoptosis analysis

MCF7 cells (1 × 10^5^ cells/ml) were seeded into 24 well plates with media and incubated for 24 h. After 24 h, cells were treated with d-limonene (0.5 mM, 1 mM, 2 mM and 4 mM) and MCF7 cells without drug treatment were used as an experimental control. After treatment of d-limonene for 24 h, the cells were harvested. Apoptosis Assay Kit (Cat no. C1062L; Abgenex, India) was used to determine apoptosis and the protocol was followed using the manufacturer’s instructions. Briefly, cells (approximately 1 million cells in 100 μl of assay buffer) were incubated with 5 μl of Annexin V FITC solution and 5 μl of Propidium Iodide (PI) nearly for 20 min at room temperature in the dark. At end of incubation, 400 μl of assay buffer was added and analyzed using flow cytometry. About 10,000 cells were acquired using FACSCanto II Flow Cytometer to detect the fluorescence (Becton & Dickinson, CA, USA). The data was analyzed using FACS Diva software.

### Cell cycle analysis

Cell cycle analysis for drug treated and untreated cells were performed using protocol that is previously described by Vanzyl et al*.*^18^. Approximately one million cells were cultured and then treated with increasing concentrations of d-limonene (0.5 mM, 1 mM, 2 mM, 3 mM, and 4 mM) for 24 h. Untreated MCF7 cell were used as control. After 24 h of treatment, cells were harvested with trypsin after centrifugation. Cell pellets were washed twice in 1XPBS. Cells were fixed using 70% ethanol followed by incubation of fixed cells at − 20 °C overnight. Ethanol was removed and cells were stained with PI for 20 min in the dark. Cells were detected by FACS Canto II Flow Cytometer (Becton & Dickinson, CA, USA) and % distribution of cells in different stages of cell cycle was calculated using FACS Diva software.

### Western blot analysis

MCF7 cells (approximately 1 × 10^6^) were seeded on a 30 mm tissue culture dish and incubated overnight till 70–80% confluence was achieved. Cells were incubated with d-limonene of different concentrations (0.5 mM, 1 mM, 2 mM, 3 mM and 4 mM) for 24 h. Cells were harvested after 24 h, suspended in modified RIPA buffer (150 mM NaCl, 50 mM Tris, pH 8.0, 5 mM EDTA, 1% 0.1% SDS, Nonidet P-40, 0.5% sodium deoxycholate) and incubated for 30 min at − 4 °C^19^. The supernatant was collected after centrifugation of the cell extract at 13,000 rpm for 20 min. Approximately 30 μg of proteins were loaded in each well, separated on 10% SDS-PAGE gels and transferred onto the PVDF membrane (Millipore Sigma, Massachusetts, U.S) as described as^20^. Primary antibodies anti-caspase 3(#14,220), anti-cleaved caspase 3(#9664), anti-Caspase 9 (#9508), anti-cleaved caspase 9 (#52,873), anti-Akt (#9272), anti-pAkt (#9271), anti- Cyclin B1 (#12,231), anti-Bcl-xL (#2764), anti-Bax (#2772), anti-CDK1(#77,055) and anti-c-Myc (#5605) were procured from Cell Signaling Technology, MA, USA. Anti- β actin (#sc-4778), goat anti-mouse IgG-HRP (#sc-2031) and goat anti-rabbit IgG-HRP (#sc-2030) were procured from Santa Cruz Biotechnology Inc., CA, USA. PVDF membranes were blocked with 5% skimmed milk and then incubated with primary antibody (1: 1000 dilutions) at 4 °C for overnight (Antibody dilution were followed by manufacture’s protocol). Blots were washed twice and then incubated with secondary antibodies (1:5000 dilutions) for 2 h. After 2 h, blot was washed and visualization of the antigen–antibody reaction were done using ECL western blotting substrate reagent (Thermo Fisher Scientific Inc, Massachusetts, U.S). β-actin expression was used to check the loading control. The densitometry analysis of all the western blot protein bands were performed by using the program Image J.

### In silico analysis

In silico analysis was performed by using a molecular docking approach. The docking was performed with d-limonene as ligand and CDK1 and Cyclin-B1 (Homo sapiens) proteins. The crystal structure of CDK1 and Cyclin-B1 proteins were downloaded from PDB website (https://www.rcsb.org/) (PDB ID: 4YC3, Resolution: 2.70 Å and PDB ID: 2B9R, Resolution: 2.90 Å). d-limonene molecule used in docking was obtained from PubChem (PubChem ID: 440,917). Hydrogen atoms were added to the d-limonene 3D structure and cleaned geometrically. The structure was converted to mol^2^ format using Chemsketch software. The proteins were prepared for docking using the Autodock Vina. Docking modes between the ligand and proteins were studied using Autodock Vina^21^. Lamarckian genetic algorithm (LGA) was employed for docking simulation. The LGA for flexible ligand–protein docking controls many degrees of freedom, providing full ligand flexibility and partial receptor flexibility. As a result, it allows individual conformations to search their local conformational space for local minima. The grid was set around the protein. A grid box dimensions of (27.966, − 5.468, 181.444 for CDK1 and − 73.783, 59.253, − 6.672 for Cyclin-B1) points in x, y and z directions were set with a grid spacing of 0.575 Å. The program was run for a total number of 500 genetic algorithm runs. Default settings were applied for all other parameters. Results from the docking describe the binding energy which is represented by binding scores of ligand on the protein of interest. Visualization, 2D- interaction plot and post-docking analysis were performed using Chimera and Discovery Studio Visualizer.

### Statistical analysis

Graph Pad Prism 6 software was used to conduct the statistical analysis. Each experiment was repeated three times for technical replicates. As biological replicates, each experiment or assay was carried out three times separately. The data shown are the mean and standard deviation of the data. To determine statistical significance, ANOVA was used. *P value ≤ 0.05, **P ≤ 0.01 and***P ≤ 0.001 denote the statistical significance.

## Results

### d-limonene inhibits cell viability of MCF7 breast cancer cells

**Figure 1:**
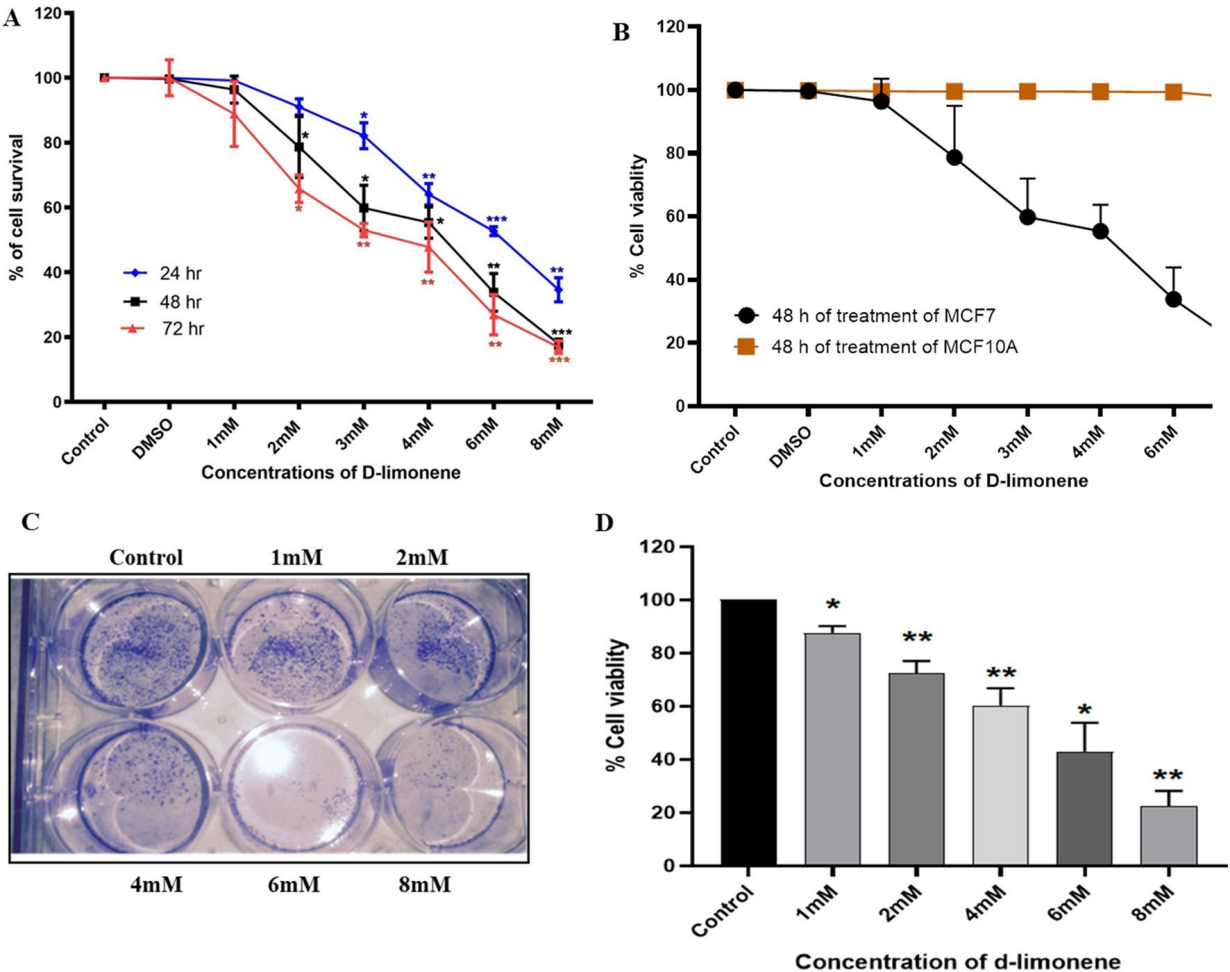
d-limonene inhibits breast cancer cell proliferation: (**A**) MCF7 cells treated with various concentrations of d-limonene as indicated in the figure and incubated for 24 h, 48 h and 72 h. The graph represents the percentage of cell viability of MCF 7 cells. (**B**) MCF10A cells treated with increasing concentrations of d-limonene for 48 h. The graph represents the percentage of cell viability of MCF10A cells. (**C**) Colony forming ability of d-limonene on MCF7 cells. (**D**) Graphical representation showing decreased colony forming ability with increasing concentration of d-limonene in MCF7 cells. Data expressed here as the mean ± SD from three independent experiments where *P-value ≤ 0.05, **P ≤ 0.01 and***P ≤ 0.001 as compared to MCF 7 cells (control).

To evaluate the cytotoxic effect of d-limonene on MCF7 breast cancer cells, survivability of cells was estimated using MTT assay. MCF7 cells were exposed to increasing concentrations of d-limonene for duration of 24 h, 48 h and 72 h and 5 μm tamoxifen as positive control. As shown in Fig. 1A, the results showed that the viability of MCF7 cells was inhibited significantly after 24 h of d-limonene treatment. Tamoxifen is also showed significant inhibition of cells proliferation as expected (Fig. S1). For d-limonene, the calculated IC_50_ value was found to be 4 mM. Additionally, cell viability was found to decrease significantly after 48 h and 72 h of exposure compared to 24 h. Further, the toxicity of d-limonene on normal breast epithelial cells MCF10A was also investigated. The obtained results suggested that cell viability remains unaltered in non-tumorigenic cells when treated with d-limonene (Fig. 1B). Comparison between the results of MTT assay of cancerous and non- cancerous cell line showed the significant selectivity of the d-limonene inhibitory effect.

To confirm this inhibitory effect, we further investigated the colony formation ability of cells in presence of different concentration of d-limonene. We performed anchorage dependent colony formation assay and observed that colony formation was inhibited significantly after 24 h of treatment of d-limonene as compared to the control group (Fig. 1C,D). All these data supported the potential anticancer effect of the natural compound d-limonene in MCF7 breast cancer cells without affecting the normal breast cells.

### d-limonene induces cell cycle arrest at G2/M in MCF7 breast cancer cells

**Figure 2:**
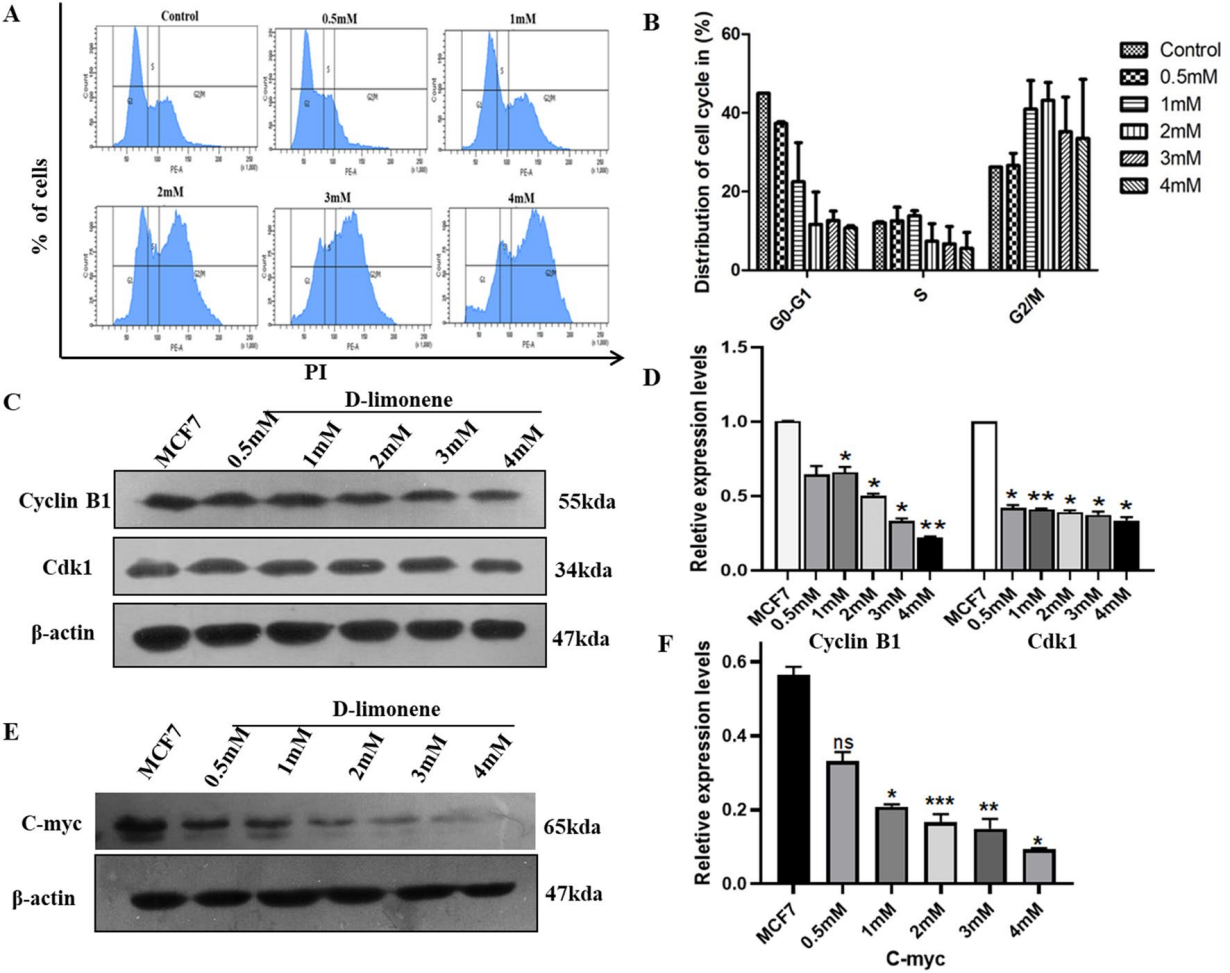
d-limonene induces G2/M arrest in breast cancer cells (**A**) Cell cycle profile was measured by flow cytometry in MCF7 cells after treatment with various concentrations of d-limonene. (**B**) Statistical analysis was done taking three independent experiments of cell cycle distribution (**C** & **E**) Expression pattern of CDK1, Cyclin B1 and c-Myc in d-limonene treated MCF7 cells checked by western blot analysis. (**D** & **F**) Relative protein levels of Cyclin B1, CDK1 and c-Myc were analyzed statistically. Data expressed here are the mean ± SD of three independent experiments and *P*-value ≤ 0.05. * and ** represent *P* ≤ 0.01 and *P* ≤ 0.001 respectively as compared to MCF 7 (control).

To further understand the anticancerous mechanism of d-limonene in MCF7 cells, we analyzed the cell cycle after 24 h of d-limonene treatment. As shown in (Fig. 2A,B) high populations of MCF7 cells were found to be arrested in G2/M checkpoint as compared to the control cells. The result was further validated by analysis of G2/M stage regulatory proteins using western blot technique. The results indicated significant decrease in the expression level of both Cyclin B1 and CDK1 as compared to control (Fig. 2C,D and Fig. S2). These results suggest that d-limonene induces cell cycle arrest successfully at G2/M phase primarily by inhibiting both Cyclin B1 and CDK1 expression. We further studied the expression of transcription factor c-Myc considering its importance in regulation of the Cyclin B1/CDK1 dependent cell cycle for arresting them at G2/M phase. The western blot analysis further showed a significant decrease in c-Myc protein expression level in d-limonene treated MCF7 cells in a dose dependent manner (Fig. 2E,F and Fig. S3). The data confirmed the involvement of c-Myc in G2/M phase cell cycle arrest induced by d-limonene.

### d-limonene stimulates the apoptosis in MCF7 breast cancer cells

**Figure 3:**
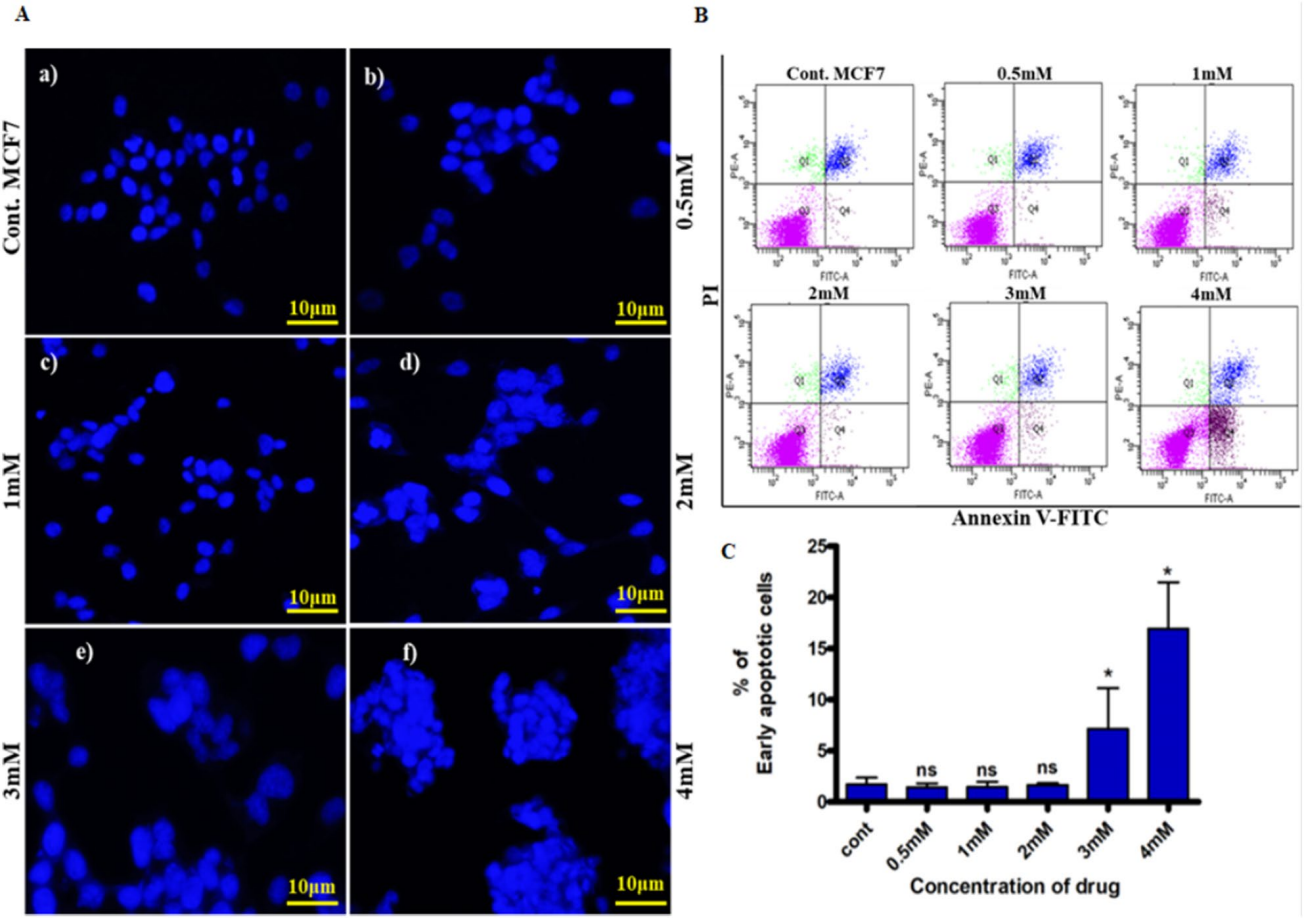
d-limonene induces apoptosis in breast cancer cells. (**A**) MCF 7 cells stained with DAPI to detect the apoptotic nuclei after 24 h of d-limonene treatment (200× magnification). (**B**) Apoptotic cells percentage was analyzed in d-limonene treated MCF7 cells using Annexin V-FITC / PI double staining method (**C**) Percentage of apoptotic cells was analyzed statistically. Data expressed here as the mean ± SD from three independent experiments where *P-value ≤ 0.05 as compared to control.

d-limonene showed inhibition of cell proliferation through morphological changes like cell shrinkage as observed microscopically (data not shown). The observation indicated to the induction of apoptosis in MCF7 cells. Hence, we further analyzed the role of d-limonene in inducing apoptosis using DAPI staining. The results showed that there was an increase in apoptotic nuclei with increasing concentrations of d-limonene. Nuclear fragmentation and condensation of chromatin were also observed at 4 mM concentration in d-limonene treated cells (Fig. 3A). Further validation was done by flow cytometry analysis through Annexin V and PI staining. The analysis indicated an enhanced rate of early apoptotic cells upon d-limonene treatment. A gradual increase in percentage of apoptotic cells with increasing concentration of d-limonene was also observed (Fig. 3B,C).

### d-limonene regulates the level of apoptotic proteins in MCF7 breast cancer cells

**Figure 4:**
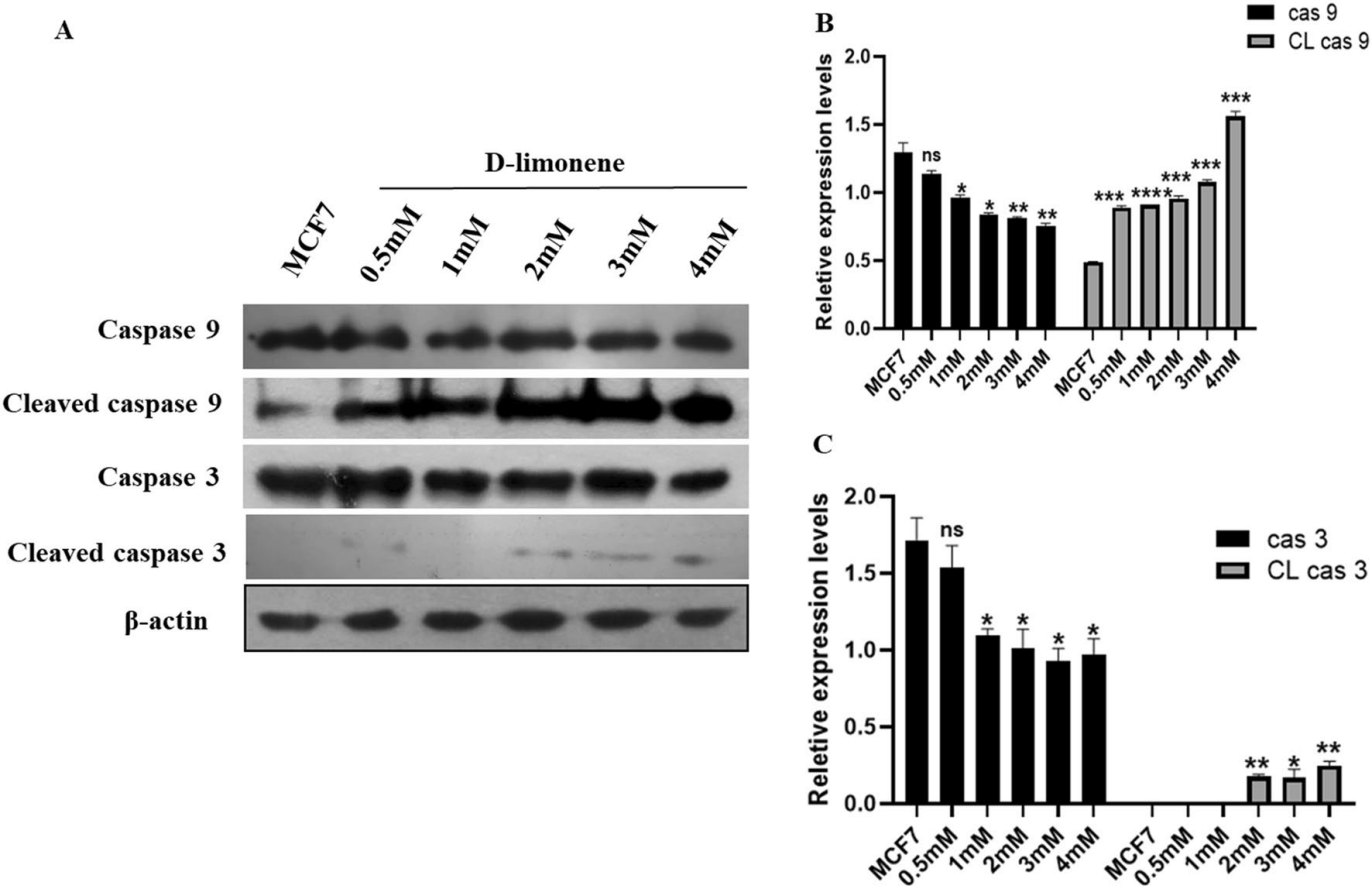
d-limonene regulates the apoptosis related protein in MCF7 breast cancer cells. (**A**) Expression pattern of molecules relevant to apoptosis detected by western blotting. (**B** & **C**) shows the relative expression levels of caspases and cleaved caspases. Data expressed as the mean ± SD from three independent experiments where *P-value ≤ 0.05, **P ≤ 0.01 and***P ≤ 0.001 as compared to MCF 7 cells (control).

Based on the flow cytometry data, we observed that d-limonene has been found to bring about apoptosis efficiently in MCF7 cells. So, we next explored the role of caspases, the executioner molecules of apoptosis by western blotting (Fig. 4A and Fig. S4). In comparison to the control, the level of expression of cleaved caspase 9 proteins and cleaved caspase 3 protein were significantly upregulated after d-limonene treatment for 24 h in MCF7 breast cancer cells (Fig. 4B,C). The results indicated the induction of apoptosis by d-limonene via apoptotic signaling pathway mediated by mitochondria in MCF7 breast cancer cells.

### d-limonene affects the Mitochondrial Apoptotic Signaling Protein Molecules

**Figure 5:**
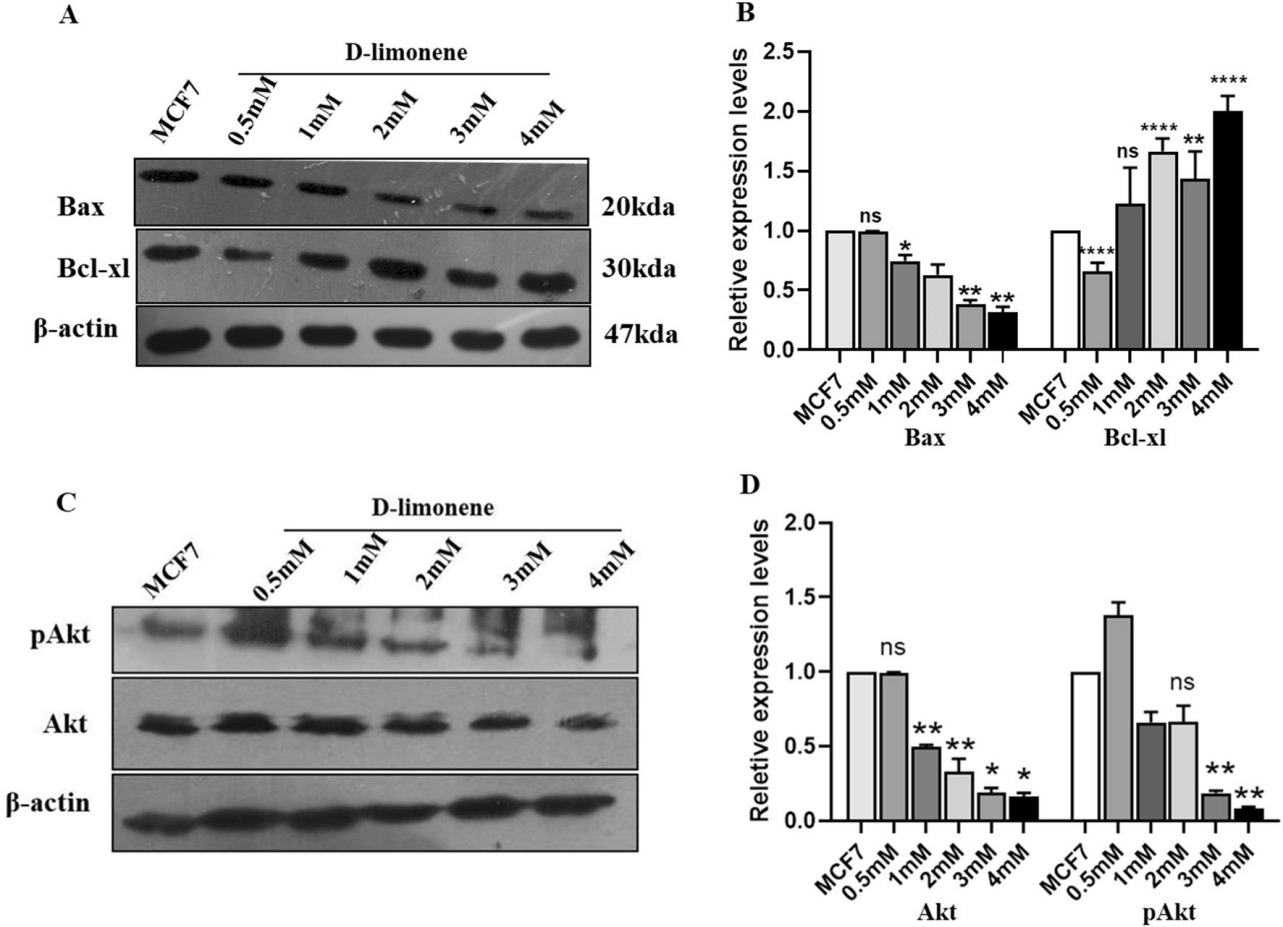
d-limonene affects mitochondrial apoptotic signaling pathway molecules. (**A**) The expression levels of proapoptotic and antiapoptotic molecules were detected by western blotting in MCF7 cells after 24 h of d-limonene treatment. (**B**) The relative levels of protein expression analyzed statistically. (**C**) Western blotting showing the expression levels of Akt and phospho-Akt in d-limonene treated MCF7 cells. (**D**) Graph showing the relative protein levels of Akt and phospho-Akt (p-Akt). Data expressed here are the mean ± SD of three independent experiments and *P*-value ≤ 0.05. * and ** represent *P* ≤ 0.01 and *P* ≤ 0.001 respectively as compared to MCF 7 (control).

As it was necessary to further verify the fact that apoptosis induction in the present study is linked to the mitochondrial cell death pathway activation by d-limonene, we investigated the expression pattern of apoptotic protein molecules by western blotting. The data indicated that the expression levels of Bax protein (pro-apoptotic) was increased significantly and there was inhibition of anti-apoptotic Bcl-xl protein in d-limonene treated MCF7 cells as compared to MCF7 breast cancer cells (Fig. 5A,B and Fig. S5). Additionally, western blot analysis also revealed that the expression of Akt and phospho Akt were deregulated in MCF7 cells, when treated with d-limonene (Fig. 5C,D and Fig. S6).

### d-limonene interact at atomic scale to influence Cyclin B1 and CDK1

**Figure 6:**
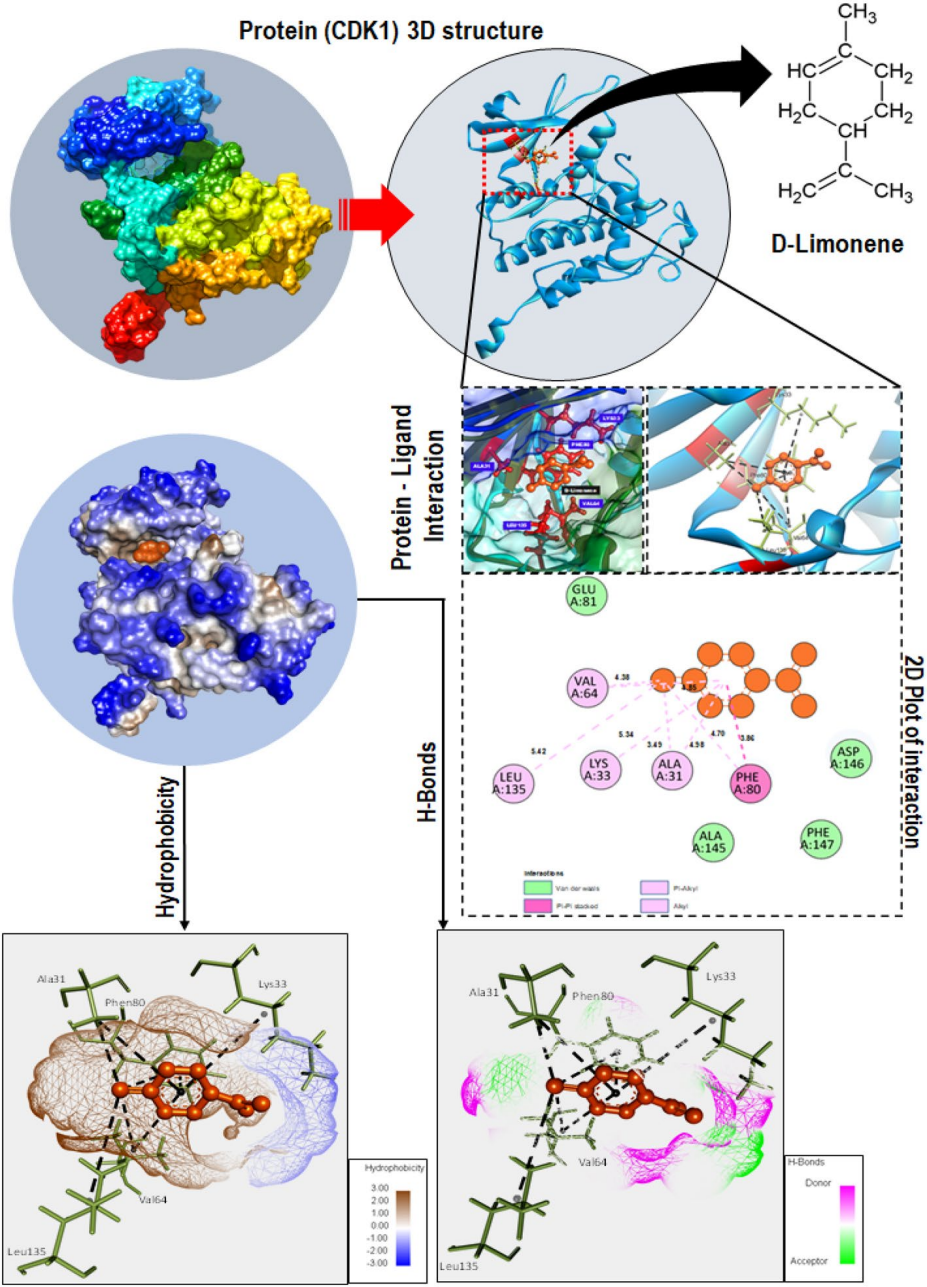
Molecular interaction of CDK1 protein (Homo sapiens) residues with d-limonene as predicted through molecular docking. The docking was performed using Autodock Vina. CDK1: Cyclin-dependent kinase 1; H-bond: Hydrogen bond. CDK1 was found to interact with d-limonene via different amino acids like valine (VAL64), leucine (LEU135), lysine (LYS33), phenylalanine (PHE80) and alanine (ALA31) through hydrogen bond and Vander wall forces through different level of binding energy.

**Figure 7:**
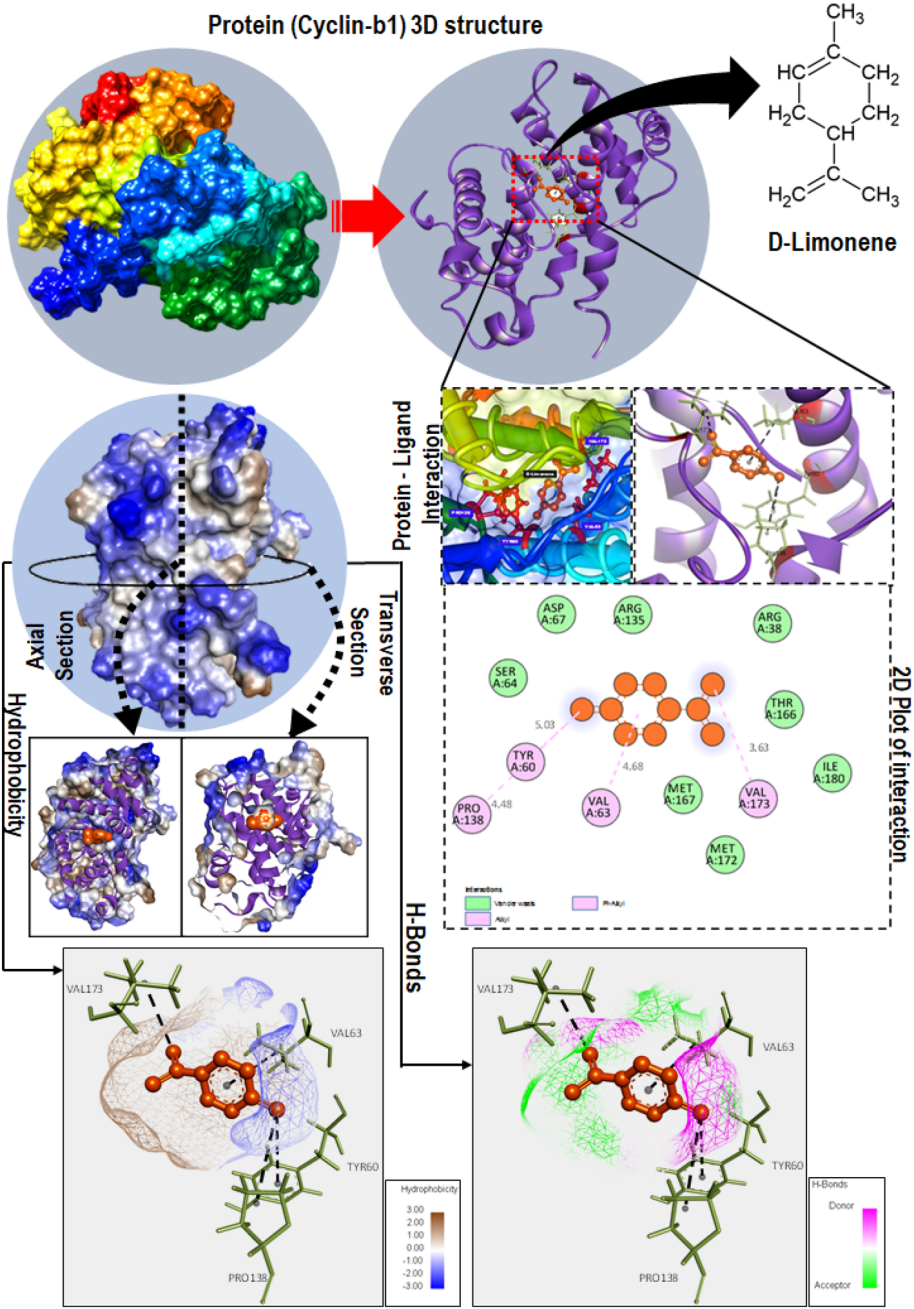
Molecular interaction of Cyclin B1 protein (Homo sapiens) residues with d-limonene as predicted through molecular docking. The docking was performed using Autodock Vina. d-limonene was predicted to interact with Cyclin B1 through tyrosine (TYR60), valine (VAL63, VAL173) and proline (PRO138) via hydrogen bond while there was also presence of Vander wall interaction through methionine (MET172, MET167), threonine (THR166), aspartic acid (ASP67) and arginine (ARG135,ARG38).

**Figure 8:**
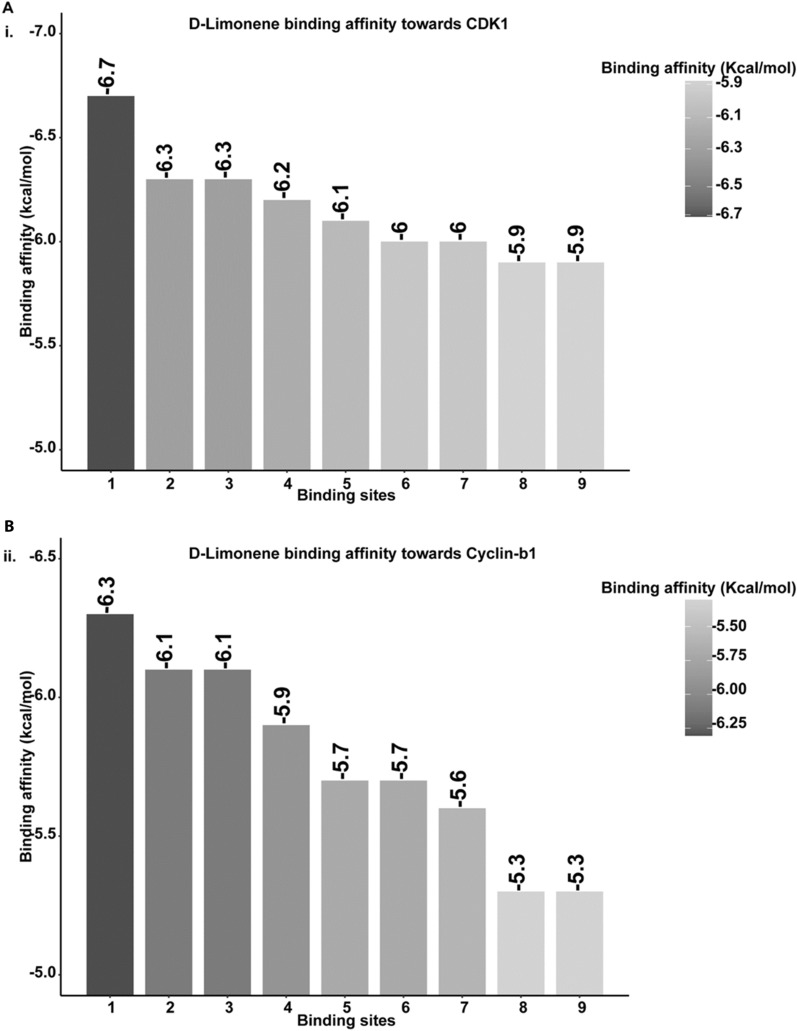
Binding energies represented by binding scores of ligand (d-limonene) on the protein of interest e. g. (i) CDK1 (ii) Cylcin-B1.

The role of d-limonene in regulating Cyclin B1 and CDK1 leading to cell cycle arrest has been indicated by their interaction at atomic and molecular level. The fact was excavated using in silico approach through molecular docking. As shown in (Fig. 6), CDK1 was found to interact with d-limonene via different amino acids like valine (VAL 64), leucine (LEU135), lysine (LYS33), phenylalanine (PHE80) and alanine (ALA31) through hydrogen bond and Vander wall forces through different level of binding energy. Similarly, d-limonene was predicted to interact with Cyclin B1 through tyrosine (TRY60), valine (VAL63, VAL173) and proline (PRO 138) via hydrogen bond while there was also presence of Vander wall interaction through methionine (MET172, MET167) threonine (THR166), aspartic acid (ASP 67) and arginine (ARG135, ARG38) (Fig. 7). The interaction predicted had been channelized through different level of binding energy as shown in Fig. 8.

## Discussion

d-limonene, one of the most common and natural monoterpene compound has been considered to have chemo-preventive activities in many types of cancers. It does not demonstrate any carcinogenic, mutagenic and nephrotoxic risk on humans^22^. Though, the results of clinical trials suggest its possible role in controlling cancer, its anticancer mechanism is not clear. Several possible mechanisms have been suggested by the researchers in recent years related to its anticancer potential of d-limonene^5,23^. Induction of apoptosis in various cancers by d-limonene has been found to be one of the most important mechanisms. In vitro studies have shown the involvement of d-limonene in inducing apoptosis^4,8,9,24^. According to a recent meta-analysis study, induction of apoptosis in various cancers by d-limonene has been found be the most important mechanism for tumor regression^25^. Various reports have also suggested that d-limonene inhibits mammary cancer in animal models^7,26,27^.

The current study explained that cell proliferation of MCF 7 was inhibited in a dose and time dependent manner by d-limonene. Particularly, we demonstrated that the inhibition of MCF 7 cells proliferation might be due to induction of the G2/M arrest in the cell cycle through Cyclin B1 deregulation leading to apoptosis and it is mediated by mitochondrial pathway. We also observed the involvement of d-limonene in regulating PI3K/Akt signaling pathways. Molecules of this pathway play a key role in cell proliferation as well as apoptosis. Further, Akt has been found to inhibit apoptosis thereby promoting cell survival^28^. Previously, Jia et al. studied the role of Akt in anticancer mechanism of d-limonene^9^. They reported the inhibition of phosphorylation of Akt by d-limonene in LS174T human colon cancer cells leading to apoptosis and affecting cell survival. In the present study, the obtained data explained the inhibition of Akt phosphorylation in MCF7 cells by d-limonene, thereby suggesting possible regulatory role of PI3K/Akt signaling pathway in cell survival process**.**

Our experiments also showed that d-limonene inhibits the growth of MCF7 breast cancer by regulating apoptosis. We further explored the possible mechanism of d-limonene in inducing apoptosis in MCF7 cells. Inside a cell, the process of apoptosis is triggered by both intrinsic and extrinsic pathways that activate the initiator and effector caspases leading to morphological and biochemical changes. Pro-apoptotic (Bax, Bak) and anti-apoptotic genes (Bcl2, Bcl Xl) play a major role in the induction of apoptosis inside a cell. d-limonene has been shown to decrease Bcl2 expression and increase Bax expression effectively in various cancers^4,8,9,24^. Jia and the group have confirmed that d-limonene is responsible for the activation of the intrinsic mitochondrial apoptotic signaling pathway in LS174T colon cancer cells. It has been shown to induce PARP cleavage along with upregulation of Bax and downregulation of Bcl2 expression in these cells^9^. Similar results were also reported in our experiments that are down regulation of Bcl Xl and upregulation of Bax in MCF7 cells by d-limonene.

Caspases (both initiator and executioner) play a major role in executing the apoptosis mechanism. The caspase – cascade activation mediated by mitochondria is initiated by activation of Bax and Bcl2 proteins inside mitochondria by external stimuli. Cytochrome C is released to the cytoplasm activating cascades of caspases which leads to apoptosis. Studies have indicated that d-limonene induces apoptosis via mitochondria mediated signaling pathways. Previously Jia et al. had reported that d-limonene initiated the activation of caspases along with an increase in the cleavage maturation of caspase 9 and 3 in human colon cancer cells^9^. Activation of caspase 8 in HL-60 human leukemia cell lines by d-limonene has also been reported^4^. A recent study on human bladder cancer cells indicated that d-limonene increases the expression pattern and activated the cleavage of caspase 3, 8 and 9. In our study, we also found that treatment of d-limonene also triggers apoptosis in MCF7 cells by the activation of caspase 9 and 3 conforming its significance^8^.

Checkpoints are the regulators of the cell cycle that allow the cells to enter the next phase. The Cyclin B1 /CDK1 complex is an important regulator of G2/M transition and it phosphorylates and dephosphorylates many proteins before the cells enter into mitosis^29^. It is synthesized in abundance, when the cell crosses through the G2/M check point and allows it to enter the M phase^30^. As per the previous studies, the expression level of CDK1/Cyclin B1 complex decreases when the cells undergo G2/M arrest^31^. Interestingly, our data also describes a decrease in both the Cyclin B1 and CDK 1 expression in MCF7 cells, when treated with d-limonene for 24 h. In concordance with our results, Yuan et al. have found that depletion of Cyclin B1 in malignant cells can inhibit proliferation and activate apoptosis induction in human tumor cells^12^. d-limonene has been reported to induce G2/M cell cycle arrest in T24 human bladder cancer cells recently^8^. Moreover, we also observed the induction of G2/M arrest in MCF7 cells when treated with d-limonene. In addition, we further report for the first time about the down regulation of both CDK1 and Cyclin B1 expression that might have been triggered by d-limonene leading to G2/M arrest in MCF7 cells.

It is well known fact that transcription factor c-Myc plays a significant role in regulating both cell proliferation and cell death^32^. In our study, we also observed dose-dependent down regulation of c-Myc in MCF7, when cells were treated with increasing concentrations of d-limonene. Previous report also explained that over expression of c-Myc oncogene in N-nitrosodiethyleamine induced liver cancer in AKR mice was down regulated significantly by d-limonene^33^. c-Myc oncogene has been described to induce Cyclin B1 promoter^34,35^. Recently as suggested by Yang et al., c-Myc was found to regulate G2/M cell cycle progression. In another study it was also found to be dependent on CDK1/Cyclin B1 expression in Raji cells via acetylation of Histone H4^36^. They observed that decrease in c-Myc expression resulted in G2/M arrest significantly. Myc has been found to stimulate cell cycle progression through CDK1 activation^37^. All these results support that c-Myc might be regulating cell cycle arrest triggering apoptosis induced by d-limonene in MCF7 cells.

The experimental analysis confirmed the action of d-limonene on cell cycle through their influence on CDK1 and Cyclin B1 proteins. The phenomenon can be attributed to the interaction of d-limonene with CDK1 and Cyclin B1 at molecular and atomic levels to influence their structural and functional activities. Hence, an in silico approach was taken through molecular docking analysis to understand the interaction of d-limonene with these proteins. The docking analysis showed the interaction of d-limonene with Cyclin B1 via amino acid like tyrosine, valine and proline. Similarly, CDK1 was found to interact with d-limonene through phenylalanine, lysine, valine and alanine. The interaction analysis indicated the influential effect of d-limonene on the structure of these proteins regulating their functionality.

**Figure 9:**
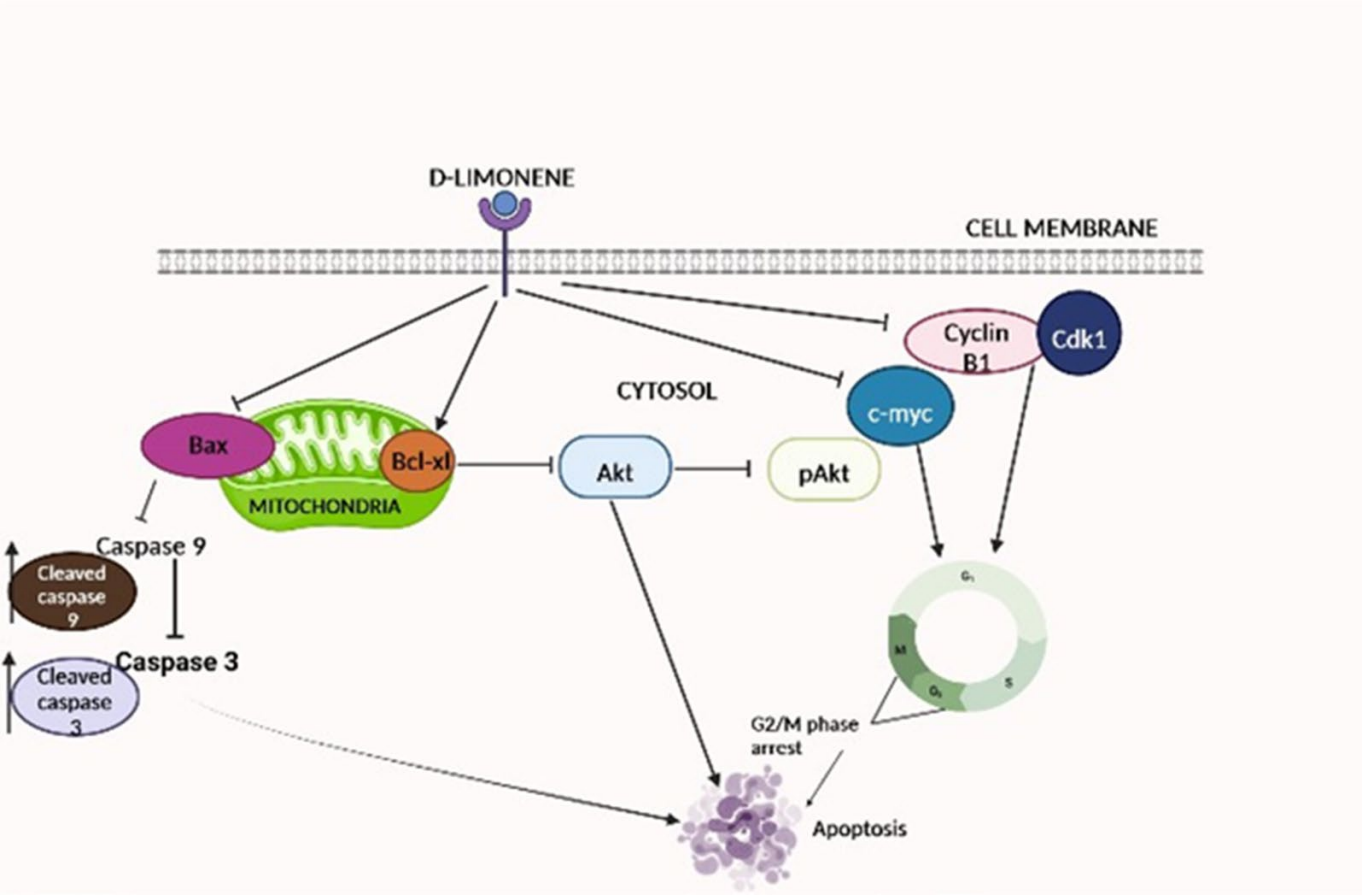
Schematic presentation of the proposed model for anticancerous mechanism of d-limonene in MCF7 breast cancer cells. The diagram indicates initiation of apoptosis by d-limonene regulating apoptosis related molecules leading to G2/M arrest eventually inhibiting cell proliferation.

It is of utmost importance to comprehend the link between cell proliferation and apoptosis for the development of new therapeutic strategies of cancer. The current study suggests that d-limonene inhibits proliferation of MCF7 breast cancer cells very effectively. Convincingly, the results also demonstrated induction of cell cycle arrest by d-limonene at G2/M phase by inhibiting Cyclin B1 and CDK1 further leading to apoptosis. The pathway can be caricatured as shown in Fig. 9. So, the failure of d-limonene treated MCF 7 cells to initiate the G2-M transition might be related to down regulation of Cyclin B1 expression. As per the results, c-Myc might also be playing an important role in this process which requires further investigations.

## Conclusions

The present study convincingly revealed that d-limonene inhibits the growth of MCF7 breast cancer cells and arrest the cell cycle at the G2/M phase by inhibiting Cyclin B1/ CDK1 complex through their influential effect on structure and function leading to induction of apoptosis. Additionally, the finding strengthens the anti-tumor potential of d-limonene suggesting its use as a chemotherapeutic and chemo-preventive drug in the treatment of breast cancer, particularly in tumors associated with Cyclin B1 and c-Myc over expression. Further, detailed analysis in this regard will definitely help in the breast cancer treatment strategies. We are also looking forward to conducting further investigations to find out the major cellular event taking place during the process of apoptosis induction after d-limonene treatment.

## Supplementary Information

Supplementary Information.

## Data Availability

All data generated or analyzed during this study are included in this published article and its supplementary information files.
