# Supplementary material for: Proximal discrepancy in intrinsic atomic interaction arrests G2/M phase by inhibiting Cyclin B1/CDK1 to infer molecular and cellular biocompatibility of d-limonene
Source: Sci Rep. 2022 Oct 28;12:18184. doi: 10.1038/s41598-022-21364-4 (PMC9616896; doi:10.1038/s41598-022-21364-4)
Supplement: Supplementary file 1 — Supplementary Information. [file 41598_2022_21364_MOESM1_ESM.docx]

**Supplementary Data**

**Proximal Discrepancy in intrinsic atomic interaction arrests G2/M phase by inhibiting Cyclin B1 / CDK1 to infer molecular and cellular biocompatibility of D-limonene.**

Deepa Mandal^1^, Paritosh Patel^1^, Suresh K. Verma^1^, Bikash Ranjan Sahu^1^, Tithi Parija^1*^

^1^School of Biotechnology, KIIT Deemed to be University, Bhubaneswar, Odisha, 751024, India.

^*^Corresponding author: Dr. Tithi Parija, Assistant Professor, School of Biotechnology, KIIT Deemed to be University, Bhubaneswar, Odisha, 751024, India, Tel: +91-9437167375

E-mail: [tithi.parija@kiitbiotech.ac.in](mailto:tithi.parija@kiitbiotech.ac.in)

**Figure S1: Cell viability (MTT assay)** **of positive control:** Cytotoxic effect of Tamoxifen (positive control) and d-limonene on MCF7 cells after the treatment of 24h. The graph represents the percentage of cell viability of MCF7 cells. Data expressed here as the mean ±SD from three independent experiments.


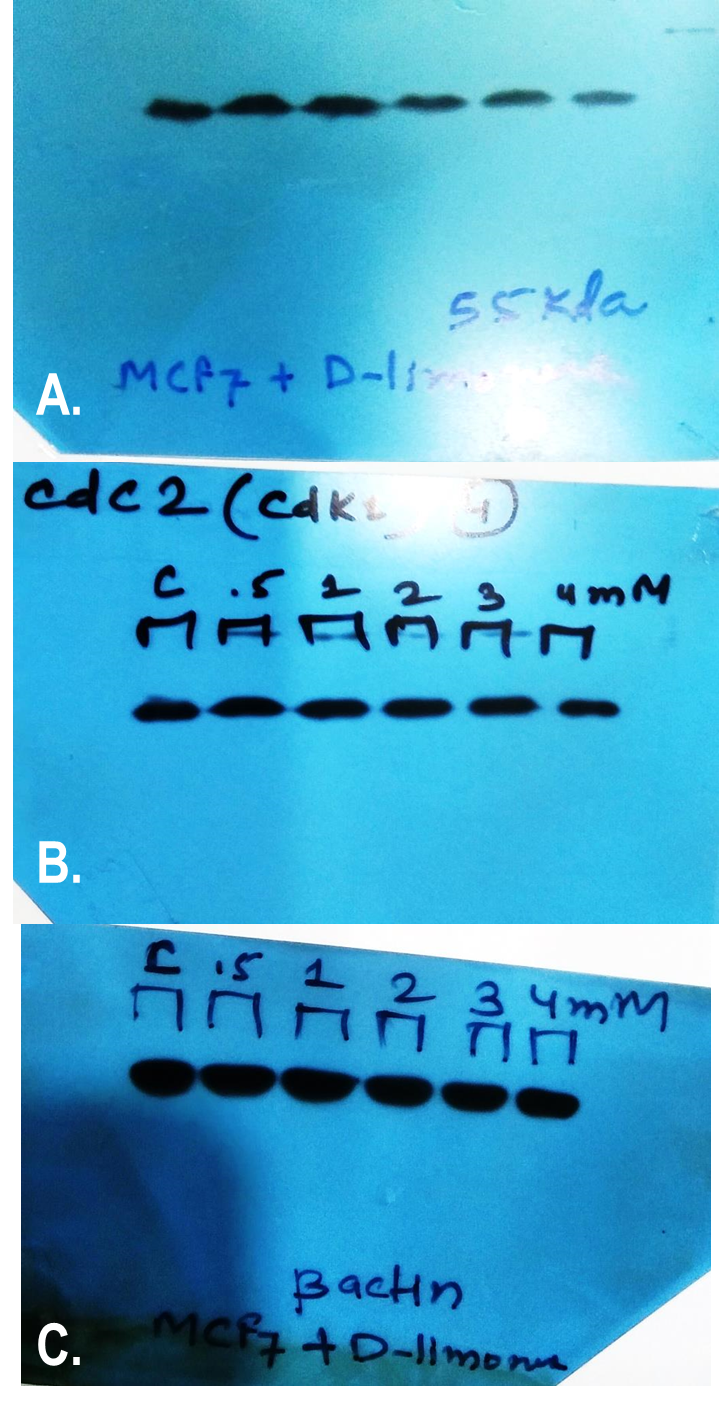


**Figure S2:** Western blot analysis for different protein expressions interpreting the induction of G2/M phase arrest in breast cancer cells by D-limonene.(A) Cyclin B1 (B) CDK1 (C) B-actin


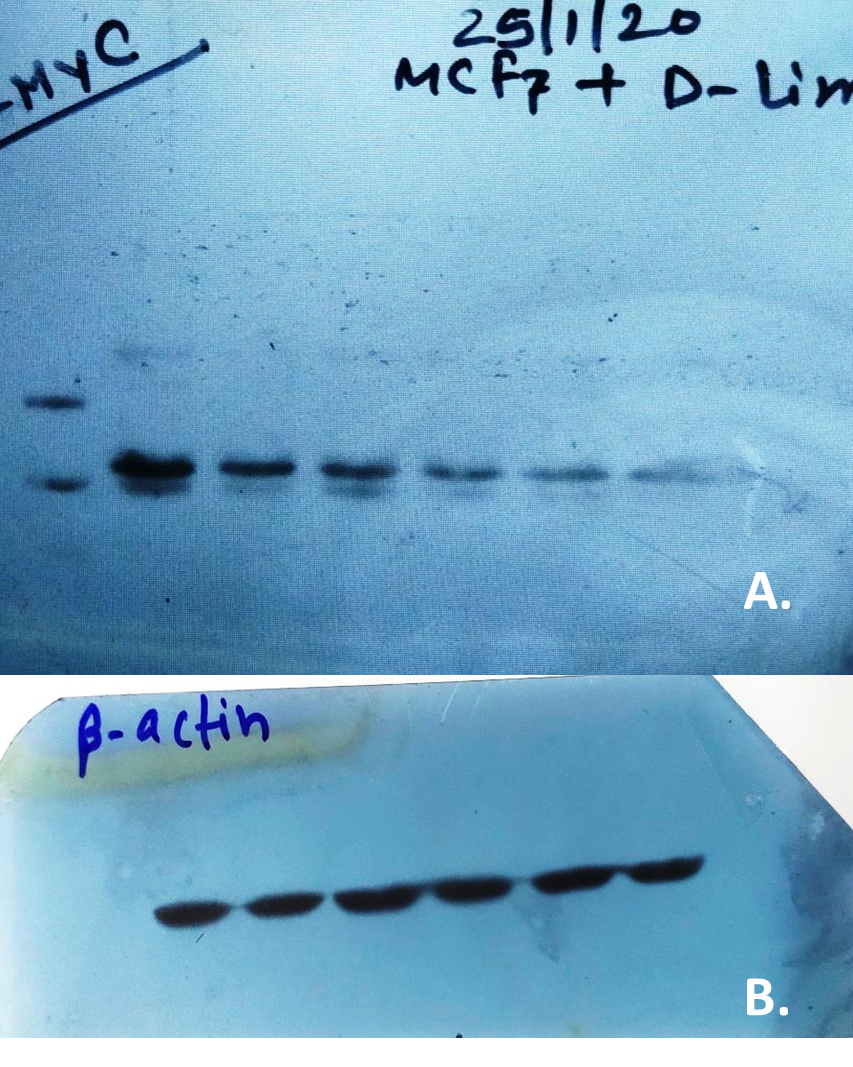


**Figure S3:** Western blot analysis for different protein expressions interpreting the induction of G2/M phase arrest in breast cancer cells by D-limonene.(A) C-MYC (B) B-actin


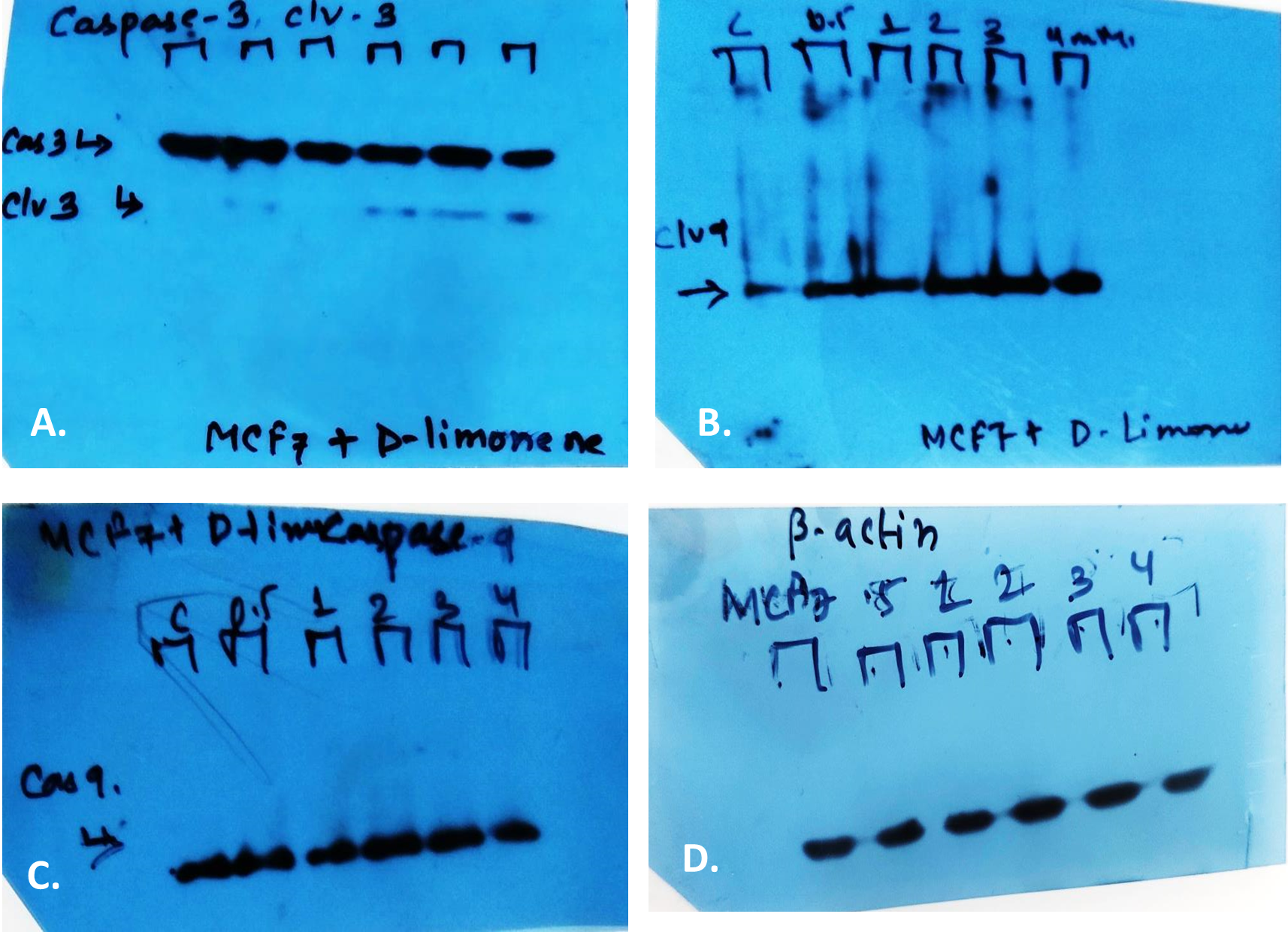


**Figure S4:** Western blot analysis for expression of proteins indicating the regulation of the apoptosis related protein in MCF7 breast cancer cells by D-limonene (A) caspase 3 and cleaved caspase 3 (B) cleaved caspase 9 (C) caspase 9 (D) B-actin.

**
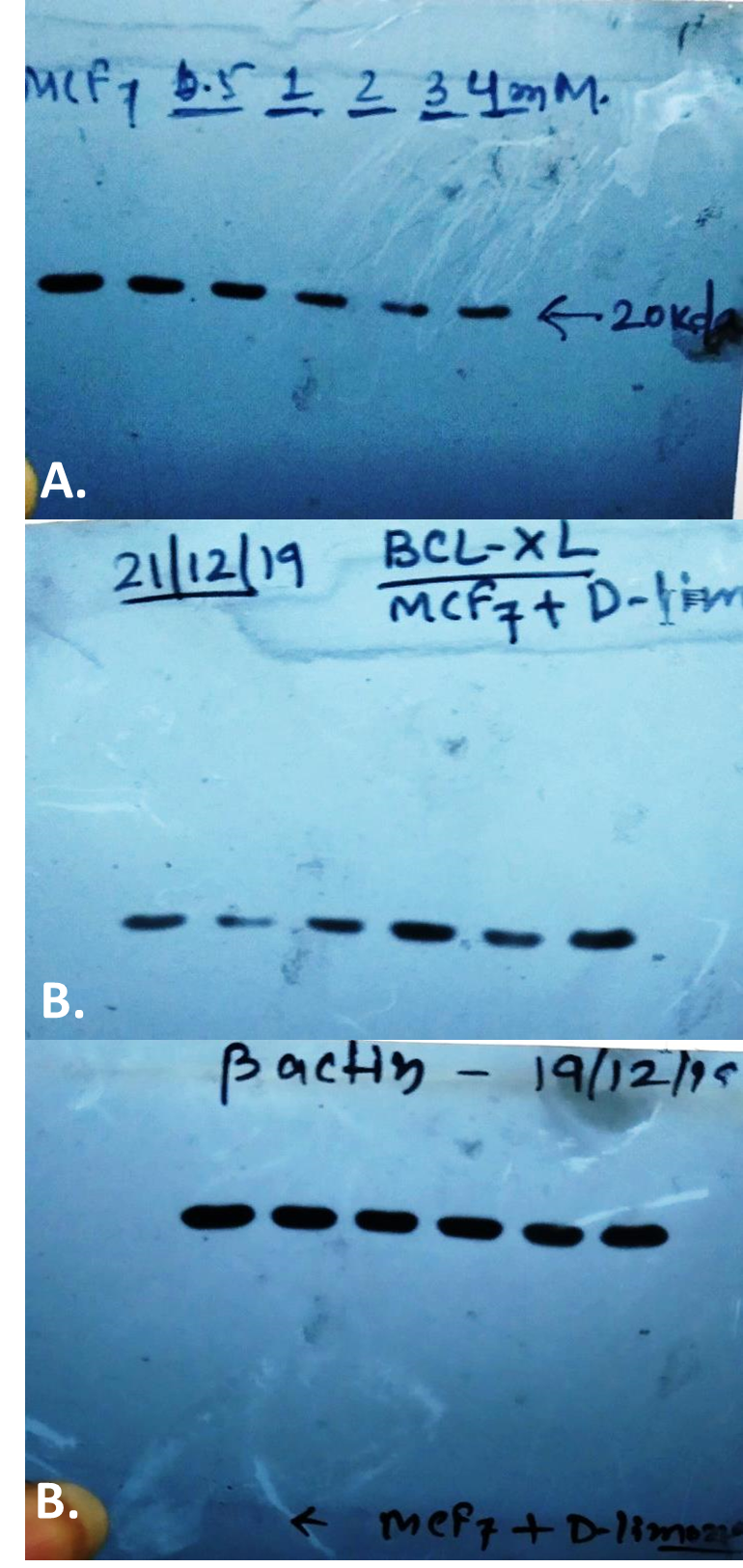
**

**Figure S5:** Western blot analysis for expression of proteins indicating the effect on mitochondrial apoptotic signaling pathway molecules in MCF7 breast cancer cells by D-limonene (A) Bax (B) Bcl-XL (C) B-actin.


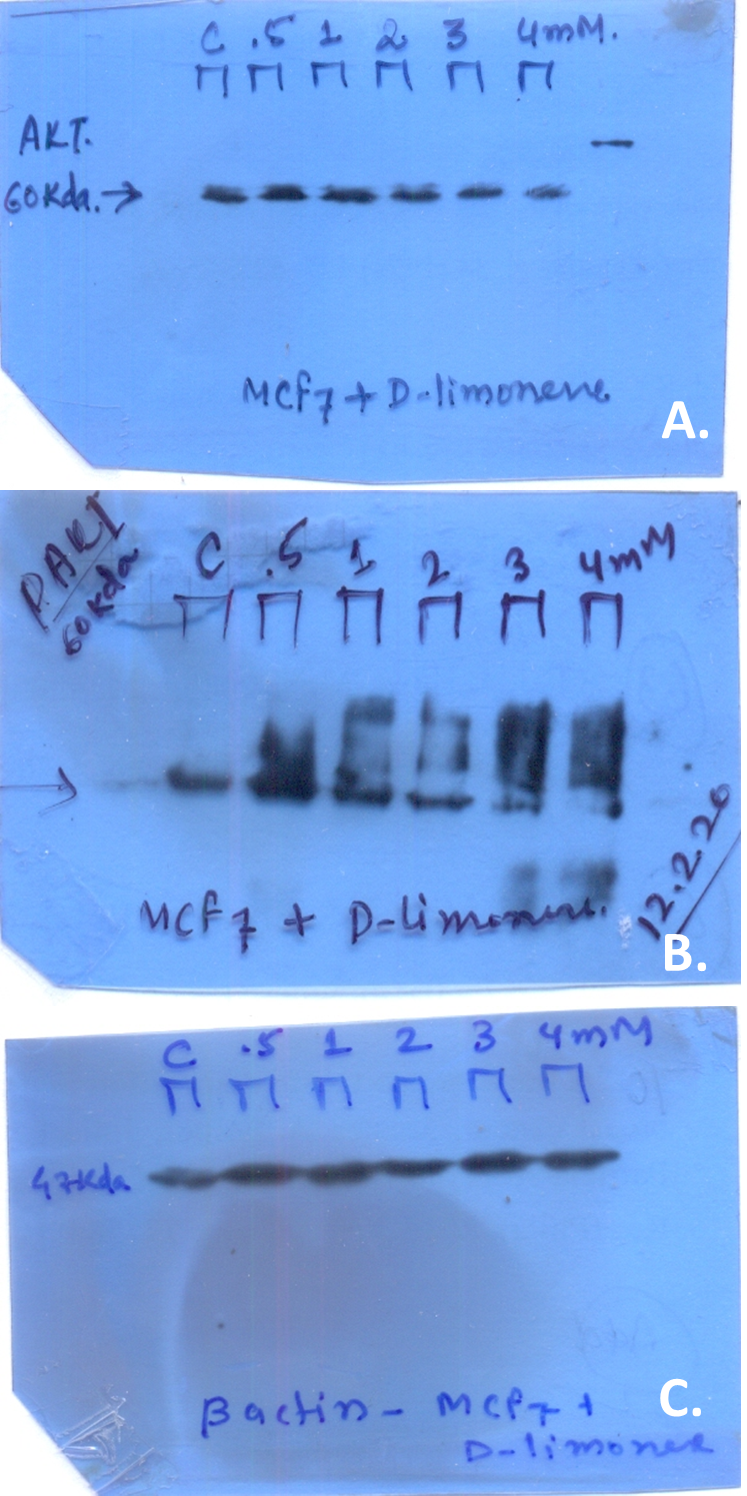


**Figure S6:** Western blot analysis for expression of proteins indicating the effect on mitochondrial apoptotic signaling pathway molecules in MCF7 breast cancer cells by D-limonene (A) Akt (B) pAkt (C) B-actin.
